# Cold Finger: Raynaud Phenomenon Following Snakebite Envenoming by Nikolsky’s Viper (*Vipera berus nikolskii*)

**DOI:** 10.3390/toxins15100598

**Published:** 2023-10-04

**Authors:** Oleksandr Zinenko, Daniela M. Durkin, Rebecca W. Carter, Brandi Ritter, Matthew R. Lewin

**Affiliations:** 1 V. N. Karazin Kharkiv University, 61058 Kharkiv, Ukraine; oleksandrzinenko@karazin.ua; 2 California Academy of Sciences, San Francisco, CA 94118, USA; daniela@dalleradurkin.org; 3 Ophirex, Inc., Corte Madera, CA 94925, USA; rebecca@ophirex.com (R.W.C.); brandi@ophirex.com (B.R.)

**Keywords:** snakebite, *Vipera*, vasospasm, Raynaud phenomenon, long-term disability, chronic manifestations of snakebite, return to duty, Nikolsky’s viper, Ukraine, Kharkiv

## Abstract

A field biologist was bitten by a female Nikolsky’s viper (*Vipera berus nikolskii*) in Kharkiv Oblast, Ukraine. Two months later, the patient began to experience cold-induced vasospasm of the affected digit diagnosed as acquired Raynaud phenomenon. The patient had more than 30 occurrences during the single winter following the bite, but the signs and symptoms of Raynaud phenomenon disappeared with the end of winter. This report describes the case and puts it into context with the literature on the topic of toxin-induced peripheral vasospastic disorders and their potential importance in snakebite envenoming.

## 1. Case Report

**Figure 1:**
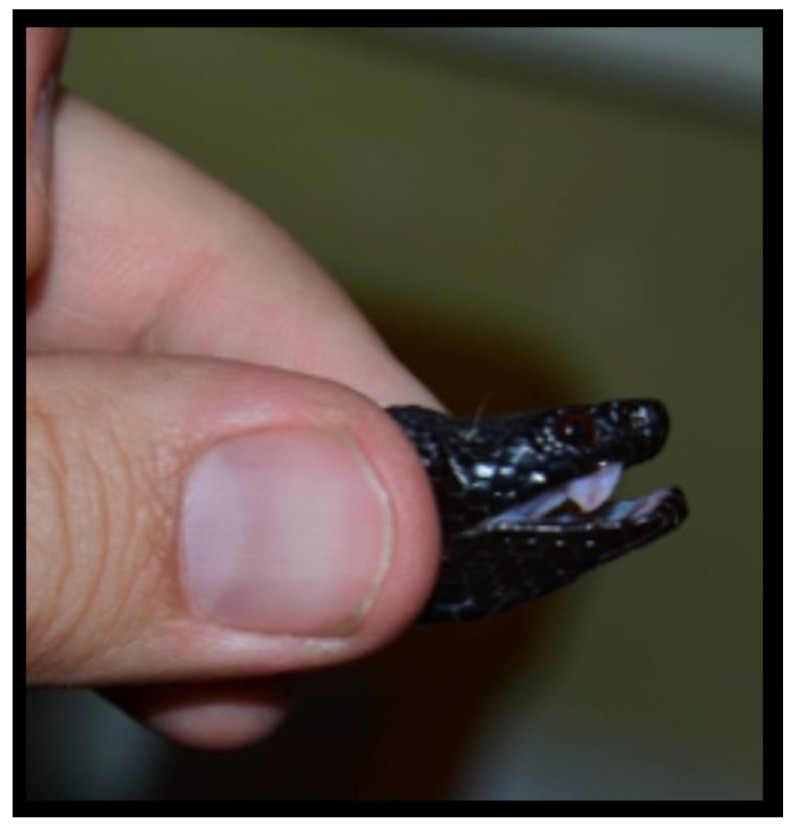
Female *V. berus nikolskii* that bit the patient.

**Figure 2:**
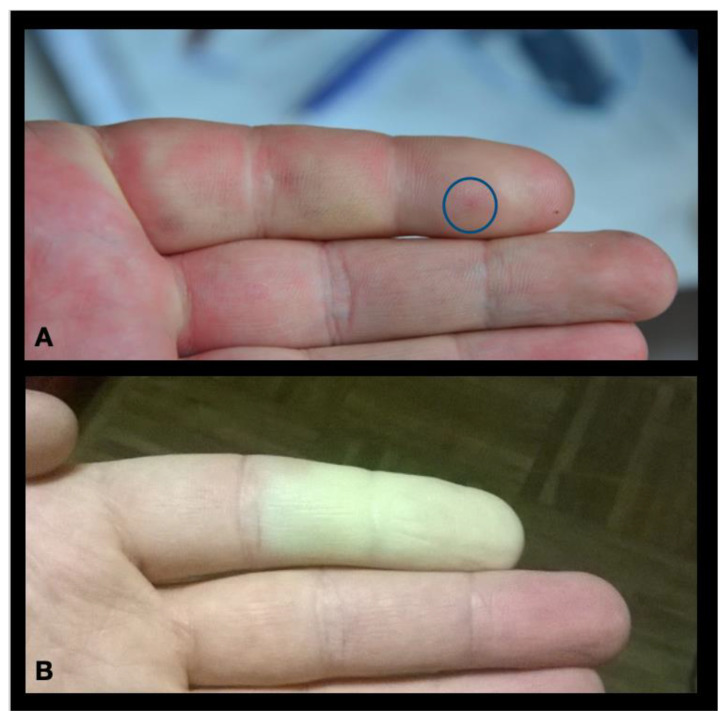
(**A**) Single fang mark (blue circle) in the pulp of patient’s distal phalanx minutes after the bite by *V. berus nikolskii* (inset) with attendant erythema and swelling as demonstrated by loss of superficial skin landmarks. (**B**) Two months following the bite, the patient began exhibiting signs and symptoms pathognomonic for secondary Raynaud phenomenon that lasted approximately one year but has not reappeared in the seven years following the bite.

**Figure 3:**
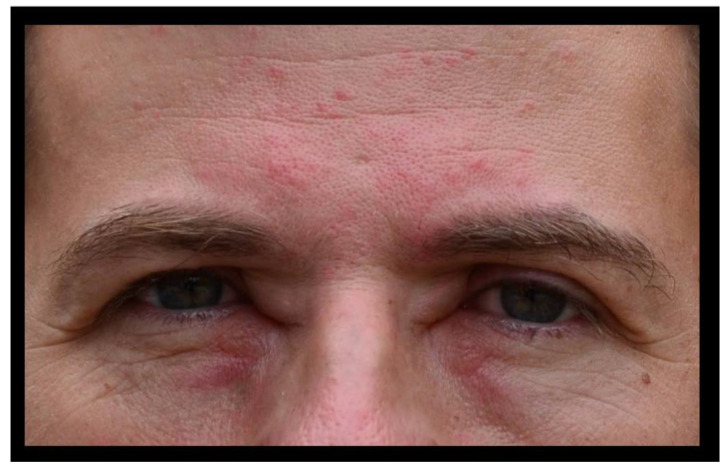
Urticaria of midface and forehead following envenoming.

During a field study, an otherwise healthy 37-year-old male biologist was handling a large adult female *Vipera berus nikolskii* (Nikolsky’s Viper) (Figure 1) when he was bitten on the second digit of his non-dominant hand by one fang (Figure 2A). He immediately experienced “stinging pain that did not radiate” at the site of the bite and a reflexive attempt was made to suck out the injected venom. Within a few minutes, typical signs and symptoms of an allergic reaction developed: itchiness of skin remote from the affected finger—such as in the patient’s inner canthi—followed by lacrimation, rhinitis, stuffed nose, widespread urticaria, and pronounced facial edema (Figure 3). At this time, the bitten finger became discolored and swollen, and edema spread proximally to the wrist and forearm. Diarrhea and generalized weakness developed around 30 min after the bite. Self-administered treatment consisted of 20 mg of loratadine delivered orally. Once in a local emergency department, the patient received intramuscular injections of 8 mg dexamethasone and 20 mg chloropyramine (Suprastin^®^). The maximum extent of pain occurred over a period of 12 h following the bite, and weakness was largely resolved by 24 h. Pain and tenderness in the skin and lymph nodes gradually subsided within two days’ time. Swelling was extant for 24 h, and normal flexion of the bitten finger was restored after 48 h. The initial treatment appeared to address the acute episode, and no antivenom was administered. No laboratory studies were undertaken.

For two months after the initial rapid recovery from the acute episode, the bitten finger and the upper extremity had a completely normal function. However, following brief mild-cold exposure on a fall day, the patient unexpectedly experienced blanching and loss of sensation of the affected finger, distal to the proximal interphalangeal (PIP) joint. The pallor color and absence of sensation lasted for approximately 1 h and disappeared after warming. He experienced similar episodes throughout the first winter following the bite, a total of approximately 30 episodes. Notably, the episodes did not reappear in the following year’s winter, approximately fourteen months after the bite and twelve months after the first ischemic episode, and they have not reappeared since.

The patient had previously been exposed to lyophilized Nikolsky’s viper venom in the laboratory and experienced allergic reactions. This was likely induced by the small, aerosolized particles as the reactions solely occurred when he did not wear a mask. The patient had no personal nor family history of Raynaud phenomenon nor of autoimmune disease.

## 2. Discussion

### 2.1. Raynaud Phenomenon

Raynaud phenomenon was first described by Maurice Raynaud in 1862 [1]. Clinically, Raynaud phenomenon (synonyms: Raynaud’s syndrome, secondary Raynaud’s) typically manifests itself as reversible tri-phasic color changes and numbness of the digits, ears, and/or nose [2]. Consensus has been achieved regarding the final common pathway as excessive vasospasm in response to stress, temperature, certain drugs, or toxins [3].

The clinical characteristics and episodic nature of this patient’s ischemic episodes are consistent with the diagnosis of Raynaud phenomenon. Furthermore, the absence of personal and family history of Raynaud phenonomenon and autoimmune disease, plus the location of vasospastic episodes occurring solely at the site of the bite, are supportive of venom-induced Raynaud phenomenon.

### 2.2. Venom-Induced Raynaud Phenomenon in English-Language Medical Literature

Only a few cases of venom-induced Raynaud phenomenon and Raynaud-like phenomena have been reported in the medical literature (Table 1). In the two cases induced by snakebite, extremital gangrene was observed as a preterminal or pre-amputation event. In Anuradhapura, Sri Lanka, the clinical progression of seven patients bitten by hump-nosed vipers (e.g., *Hypnale hypnale*) were monitored until recovery or death [4], one of whom was reported as having Raynaud phenomenon leading to ascending gangrene of distal limbs. However, the patient was also reported to have disseminated intravascular coagulation (DIC) along with renal cortical necrosis (RCN). It is possible, if not likely, that DIC contributed to the distal limb gangrene. An assessment of the vasculature postmortem was not reported, and the definitive etiology of the vascular event remains unclear. In another report [5], a 46-year-old male was bitten on the right foot by a Russell’s viper (*Daboia russelii*) and in the following days developed ischemic signs and symptoms on both upper and lower extremities, including gangrene. In situ thrombosis, discrete embolic events, and DIC were likely aspects of the differential and the ultimate etiology in this case remains unclear.

Overall, the sparse literature with respect to venom-induced Raynaud phenomenon highlights the fact that diverse mechanisms may underlie various ischemic events that may be mistakenly attributed to Raynaud phenomenon. Such mechanisms include DIC, thromboembolic events, hyperviscosity syndromes, and venom-induced consumptive coagulopathy (VICC). While there are many possible pathways to acquire Raynaud phenomenon [11,12], the final common pathway describing the clinical syndrome of severe, cold-induced digital vasoconstriction appears to be consistent across diverse triggers: A decrease in regional body temperature (e.g., hands or digits) increases the activity of sympathetic nerve fibers and stimulates the release of norepinephrine. Norepinephrine induces constriction by activating post-junctional alpha adrenoreceptors located on smooth muscle cells, resulting in similar local effects observed when injecting epinephrine directly into subcutaneous tissues [13,14,15].

### 2.3. Vipera berus Venom

*V. berus nikolskii* is one of approximately six members of the *V. berus* complex spanning Europe and Eurasia [16]. Its venom and that of related species and subspecies are relatively well studied. Briefly, venom phospholipase A2s (sPLA2s) primarily from the D49 subgroup are the main enzymes, composing 65% of the venom’s dry mass, and serine proteases are the second most abundant, composing 19% of its dry mass. There are insufficient data in humans, and no specific animal model of Raynaud phenomenon that would implicate a specific venom component within *Vipera* or other venoms, so this should be an area of attention if future cases are reported and hypothesis generating patterns emerge.

Generally, the main signs and symptoms of *V. berus* bites are local edema and pain [16], but snakes from this group are known to cause neurotoxicity in young children and, more commonly, significant systemic toxicity in older adults [17]. Other European *Vipera* species are known to cause life-threatening neurotoxicity in adults as well as children [18], showing the diversity of toxicity in this less famous, but at times dangerous and medically vexing viper group. In addition to this case, some significant long-term sequelae have occurred in military personnel. For example, a soldier in Europe was bitten in the forehead by a *V. berus* on the ground, and after the acute facial swelling and petechiae resolved—several days’ time—the patient was left with permanent ptosis and myosis (Horner’s syndrome) [19]. As with this patient, even where envenomings are rarely lethal or totally debilitating [19,20], long-term sequelae very much exist.

### 2.4. Raynaud Phenomenon, Snakebite and Occupational Medicine

While it does not appear that Raynaud phenomenon has been described as an occupational hazard of snakebite, snakebite is a well-described occupational hazard of agricultural work, filed biology and military professions. From an occupational medicine standpoint, Raynaud phenomenon can be debilitating. Its development should prompt an evaluation for capability to return to or continue regular duty. For example, US military members who acquire Raynaud phenomenon caused by military service are evaluated for their capability to continue military service, but as a preexisting condition, it might be considered disqualifying [21,22,23,24]. Considerations for return to work that might be generally applicable to other occupations include, but are not limited to, the member’s assigned job, level of disability, applicable safety regulations, and the impact on the member’s overall health. In some cases that do not resolve, workers can be retrained into a different job where the medical condition does not have a significant impact on performance.

## 3. Conclusions

Chronic/long-term sequelae of snakebite are not nearly as well studied as acute injury and short-term recovery. Until long-term complications resulting from snakebite are reported on and studied more robustly, there will be little awareness of or systematic research following the acute phase of disease. Raynaud phenomenon and ischemic phenomena, long-functional recovery times, chronic pain syndromes and the like are probably much more common than reported and associated with severe economic damages from snakebite for which greater awareness has been manifest in the past decade [25,26,27,28,29,30,31]. More longitudinal studies are necessary to understand both the full impact of this neglected phenomenon and the scope of extant and future therapeutic interventions [20,32].

## Figures and Tables

**Table 1:** Venom sources reported in English literature to induce Raynaud Phenomenon.

Venom	Comments
Cnidaria [6,7]	*Physalia* sp. (Man O’ War) venom contains the hemolytic protein physalitoxin a [8], which composes 28% of its protein content. It is thought to be the main contributor to the venom’s lethality and hemolytic activity.
Fish [9]	The lesser and greater weever fish (*Echiichthys draco*) are venomous species primarily found in Atlantic and North-Sea waters with sporadic occurrence throughout the Mediterranean Sea.
Swarming Hymenoptera (Bees, wasps, ants)	Reported from therapeutic use of bee sting and reported fire ant envenoming [10].
Snakebite?	Reported in association with acute embolic, necrotic or terminal events [4,5].
